# Feasibility of Robotic Rectal Surgery Using the Hinotori Surgical Robot System

**DOI:** 10.1002/wjs.70138

**Published:** 2025-10-14

**Authors:** Hideya Kashihara, Takuya Tokunaga, Toshiaki Yoshimoto, Yuma Wada, Chie Takasu, Masaaki Nishi, Yuji Morine

**Affiliations:** ^1^ Department of Surgery Institute of Health Biosciences The University of Tokushima Tokushima Japan

**Keywords:** hinotori Surgical robot system, rectal cancer, robotic surgery

## Abstract

Patients' characteristics in hinotori and dV cases.

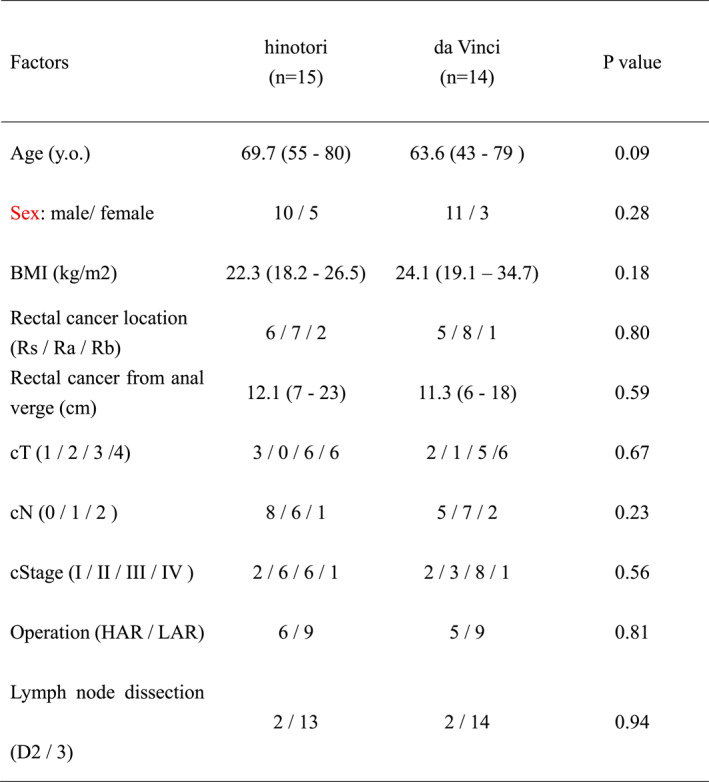

## 1 Introduction

The hinotori Surgical Robot System (Medicaroid Co. Ltd., Kobe City) is the first surgical robot to be manufactured in Japan. Since its launch in 2020, more than 600 urologic surgical procedures have been performed in Japan using this system, and the initial findings have indicated that it is safe [1]. Various types of surgical robot, including the hinotori system, have been introduced and are currently in operation. In 2023, Miura et al. reported the world's first rectal cancer surgery using the hinotori system. However, there have been no comparisons of robotic surgery using the hinotori system and the widely used da Vinci system. Here, we report the first comparison of the outcomes of robotic surgery achieved using the hinotori and da Vinci systems.

## 2 Material and Methods

### 2.1 Patients

We studied 29 patients with rectal cancer who were surgically treated between January 2023 and October 2024 (da Vinci [group D], *n* = 14) and (hinotori [group H], *n* = 15) by two surgeons with da Vinci Proctor certification. Table 1 summarizes the characteristics of the 29 patients studied, all of whom were in generally good condition. All patients in the two groups did not receive preoperative chemo and radiation therapy.

**TABLE 1:** Patients' characteristics in hinotori and dV cases.

Factors	Hinotori (*n* = 15)	da Vinci (*n* = 14)	*p* value
Age (y. o.)	69.7 (55–80)	63.6 (43–79)	0.09
Sex: Male/female	10/5	11/3	0.28
BMI (kg/m2)	22.3 (18.2–26.5)	24.1 (19.1–34.7)	0.18
Rectal cancer location (Rs/Ra/Rb)	6/7/2	5/8/1	0.80
Rectal cancer from anal verge (cm)	12.1 (7–23)	11.3 (6–18)	0.59
cT (1/2/3/4)	3/0/6/6	2/1/5/6	0.67
cN (0/1/2)	8/6/1	5/7/2	0.23
cStage (I/II/III/IV)	2/6/6/1	2/3/8/1	0.56
Operation (HAR/LAR)	6/9	5/9	0.81
Lymph node dissection (D2/3)	2/13	2/14	0.94

### 2.2 Surgical Procedure

Four robot ports and an upper right auxiliary port were placed, as used with the da Vinci surgical system in our department. The instruments used during the procedure were bipolar fenestrated forceps in the first arm of the robot, an endoscope in the second arm, monopolar curved scissors or bipolar Maryland forceps in the third arm, and Croce grasping forceps in the fourth arm. The surgical procedure was performed in the same way as with the da Vinci surgical system. The same four port with one assistant port and instrumentation was used for both platforms.

## 3 Results

There was no difference in operative time between the two groups. There was one Clavian Dindo grade 2 complication in group D (7.1%; ileus) and one Clavian Dindo grade 3 complication in group H (6.7%; anastomotic leakage). There was no difference in the duration of the postoperative hospital stay between the groups. There were also no differences in the number of dissected lymph nodes, distal margin, and all the CRMs were negative (Table 2).

**TABLE 2:** Operative and pathological outcomes in hinotori and dV cases.

Factors	Hinotori (*n* = 15)	da Vinci (*n* = 14)	*p* value
Operation time (min.)	213.7 ± 29.9	215.5 ± 24.8	0.86
Blood loss (mL)	7.3 ± 4.8	13.6 ± 11.2	0.06
Convert to laparotomy (%)	0	0	NA
R (0/1/2)	15/0/0	14/0/0	NA
Complication (CD grade 2 ≤)	1 (6.7%)	1 (7.1%)	0.95
Complication (CD grade 3 ≤)	1 (6.7%)	0 (0%)	0.33
Urinary dysfunction (%)	0	0	NA
Hospital stay (days) (median)	9.0	9.5	0.87
Mortality (%)	0	0	NA
pT (1/2/3/4)	6/3/5/1	2/4/8/0	0.28
pN (0/1/2)	10/4/1	11/1/2	0.50
pStage (I/II/III/IV)	6/6/2/1	6/5/3/0	0.51
Tumor diameter (mm) (median)	40.0	37.5	0.82
Harvested lymph nodes (median)	13.0	18.0	0.42
Distal margin (mm) (median)	25.0	30.0	0.89
CRM≤ 1 mm	0	0	

## 4 Discussion

In the present study, we have shown the feasibility of robotic rectal surgery using the hinotori Surgical Robot System through a comparison with the da Vinci Xi Surgical Robot System. Surgery performed using the hinotori system was associated with oncologic safety and a low incidence of complications. Therefore, this approach may represent a promising treatment option for rectal tumors.

The hinotori surgical robot system has several features that differ from those of the da Vinci surgical system. First, each robot arm has eight axes and the arm base angle can be specified. This operation arm moves smoothly like a human arm, reducing the collision in the sterile field, and is expected to allow the operation to proceed smoothly. Second, the surgeon's cockpit is designed with superior ergonomics, involving a flexible 3D viewer, which should reduce the burden on the surgeon and assist the precise surgery. Third, the hinotori has a docking‐free mechanism, which eliminates the need to attach the trocar to the robotic arm. This design provides ample space around the trocar and reduces the amount of tissue damage caused by excessive traction. Its points of similarity to the da Vinci system include the multi‐joint movement of the surgical arm and the method of controlling the arms by hand and using the foot unit. Thus, surgeons who are familiar with the da Vinci system should be able to acclimate to hinotori with relative ease. One limitation is that currently available instrumentation is limited with no vessel sealing or stapler devices. Future instrument development would likely change this. The hinotori system has the potential to enable preservation of the vital organs such as nerves and accurate tumor removal, thanks to its multi‐joint functionality, which permits easy maneuverability within the pelvis, the excellent 3D field of view with high resolution, and its docking‐free design, which makes it easier for assistants to operate. In the present study, the use of the hinotori system was associated with short‐term outcomes that were comparable to those achieved using the da Vinci system, and the resected specimen quality was shown to be the same between the two groups. Although there have been previous case reports of rectal surgery using the hinotori system [2], this is the first study to compare the utility of this system with that of the da Vinci system. In addition, for robot‐assisted radical prostatectomy, the hinotori system has been shown to be associated with comparable surgical outcomes to those achieved using the da Vinci system [3].

This is the first study to demonstrate the perioperative outcomes of rectal surgery using a new surgical robotic system, the hinotori Surgical Robot System, through a comparison with those associated with the da Vinci system. However, larger prospective studies are needed to demonstrate the usefulness of hinotori system for rectal surgery.

## Author Contributions

**Hideya Kashihara:** conceptualization, resources, data curation, formal analysis, investigation, methodology, writing – original draft. **Takuya Tokunaga:** data curation, methodology, supervision, writing – review and editing. **Toshiaki Yoshimoto:** data curation, formal analysis, validation, methodology. **Yuma Wada:** data curation, methodology. **Chie Takasu:** data curation, methodology. **Masaaki Nishi:** supervision, writing – review and editing. **Yuji Morine:** supervision, project administration, writing – review and editing.

## Ethics Statements

This study was approved by a suitably constituted Ethics Committee of the Tokushima University Hospital (ToCMS: 1277‐4) and was performed in accordance with the Declaration of Helsinki.

## Consent

All patients or their authorizers gave written informed consent before the operation.

## Conflicts of Interest

The authors declare no conflicts of interest.

## Data Availability

The authors have nothing to report.
